# Proteins inform survival-based differences in patients with glioblastoma

**DOI:** 10.1093/noajnl/vdaa039

**Published:** 2020-03-17

**Authors:** L C Stetson, Quinn T Ostrom, Daniela Schlatzer, Peter Liao, Karen Devine, Kristin Waite, Marta E Couce, Peggy L R Harris, Amber Kerstetter-Fogle, Michael E Berens, Andrew E Sloan, Mohammad M Islam, Vilashini Rajaratnam, Shama P Mirza, Mark R Chance, Jill S Barnholtz-Sloan

**Affiliations:** 1 Case Comprehensive Cancer Center, Case Western Reserve University School of Medicine, Cleveland, Ohio, USA; 2 Department of Medicine and Division of Hematology-Oncology, Dan L. Duncan Comprehensive Cancer Center, Baylor College of Medicine, Houston, Texas, USA; 3 Department of Medicine, Section of Epidemiology and Population Sciences, Baylor College of Medicine, Houston, Texas, USA; 4 Center for Proteomics and Bioinformatics and Department of Nutrition, Case Western Reserve University School of Medicine, Cleveland, Ohio, USA; 5 Department of Population and Quantitative Health Sciences and Cleveland Center for Health Outcomes Research (CCHOR), Case Western Reserve University School of Medicine, Cleveland, Ohio, USA; 6 Department of Pathology, University Hospitals Cleveland Medical Center, Cleveland, Ohio, USA; 7 Brain Tumor and Neuro-Oncology Center & Center of Excellence, Translational Neuro-Oncology, Department of Neurosurgery, Seidman Cancer Center, University Hospitals Cleveland Medical Center, Cleveland, Ohio, USA; 8 Translational Genomics Research Institute (TGen), Phoenix, Arizona, USA; 9 Department of Chemistry and Biochemistry, University of Wisconsin-Milwaukee, Milwaukee, Wisconsin, USA

**Keywords:** glioblastoma, mass spectrometry, proteomics, survival

## Abstract

**Background:**

Improving the care of patients with glioblastoma (GB) requires accurate and reliable predictors of patient prognosis. Unfortunately, while protein markers are an effective readout of cellular function, proteomics has been underutilized in GB prognostic marker discovery.

**Methods:**

For this study, GB patients were prospectively recruited and proteomics discovery using liquid chromatography–mass spectrometry analysis (LC-MS/MS) was performed for 27 patients including 13 short-term survivors (STS) (≤10 months) and 14 long-term survivors (LTS) (≥18 months).

**Results:**

Proteomics discovery identified 11 941 peptides in 2495 unique proteins, with 469 proteins exhibiting significant dysregulation when comparing STS to LTS. We verified the differential abundance of 67 out of these 469 proteins in a small previously published independent dataset. Proteins involved in axon guidance were upregulated in STS compared to LTS, while those involved in p53 signaling were upregulated in LTS. We also assessed the correlation between LS MS/MS data with RNAseq data from the same discovery patients and found a low correlation between protein abundance and mRNA expression. Finally, using LC-MS/MS on a set of 18 samples from 6 patients, we quantified the intratumoral heterogeneity of more than 2256 proteins in the multisample dataset.

**Conclusions:**

These proteomic datasets and noted protein variations present a beneficial resource for better predicting patient outcome and investigating potential therapeutic targets.

**Key Points**

1. Short-term and long-term glioblastoma survivors exhibit distinct protein profiles.
2. Protein abundance has a low correlation with mRNA expression in glioblastomas.
3. Glioblastomas exhibit protein abundance intratumoral heterogeneity.

**Importance of the Study**

This study uses shotgun proteomics data from multiple independent, clinically annotated glioblastoma datasets to improve our knowledge of glioblastoma biology. By illustrating the differing protein profiles of short- and long-term glioblastoma survivors, we show that proteomics is an approach that can generate meaningfully prognostic characterizations. Furthermore, by pairing proteomics with matched patient transcriptome profiling, we confirm that gene expression is a poor surrogate for protein measurement in glioblastomas. Finally, by generating a multisample glioblastoma dataset, we provide an initial evaluation of intratumoral heterogeneity at the protein level which could lead to significant insights for developments of biomarkers and targeted treatments.

Glioblastoma (GB) is the most common and most lethal malignant primary brain tumors in adults, with a median survival of 12–14 months.^1^ GBs display multiform histopathologies and manifest complex molecular aberrations that are not fully functionally characterized using genetic mutations.^2^ Histological grading of tumors may misclassify patients given the complexity and diversity of molecular alterations in GB.^3^ Most importantly, the varied nature of the disease leads to significant variability in response to standard therapy (surgery plus concurrent radiation and temozolomide).^4^ Age at diagnosis, extent of surgical resection, and preoperative Karnofsky Performance Status (KPS) are well-described prognostic factors for GB.^5^ An unmet need in routine clinical care for GB patients, as well as in drug discovery for this disease, is the development of high-throughput molecular approaches that can both classify current patients to improve clinical trial designs and identify new therapeutic targets not discernible using current genomics approaches.

Overall, problems of intra- and intertumoral heterogeneity and functional relevance create challenges for the independent validation of prognostically significant markers in GBs. Although early studies revealed unique mutational, epigenetic, and transcriptional signatures in GBs,^6–8^ these signatures have not become routinely used in clinical practice and are not applicable for all GB patients. Two prognostic biomarkers have been consistently verified in GB: isocitrate dehydrogenase 1/2 (*IDH1/2*) mutation and hypermethylation of *O*^6^-alkylguanine DNA alkyltransferase (*MGMT*). *IDH1/2* mutation is associated with an overall survival advantage, but only occurs in approximately 5–10% of GB patients and is considered to be an indicator of progression from lower grade glioma.^8,9^*MGMT* methylation produces a survival advantage by suppressing DNA repair and increasing the efficacy of chemoradiation, but it is present in only 20–40% of GB patients.^10^ More recently, a comprehensive high-throughput genomic and transcriptomic profiling of GBs demonstrated that there is no distinctive genomic or transcriptomic signature among *IDH1/2* wild-type GB patients (the majority of patients) who are long-term survivors.^11^ This finding highlights the need for improved prognostic markers among the *IDH1/2* wild-type patient population. Although the above-mentioned gene-based markers are of considerable interest, they have not translated into changes in clinical care for the vast majority of GB patients.

The use of gene expression profiling as a proxy for downstream functional activity is dependent on there being a close correspondence between mRNA expression and protein expression or activity.^12^ Proteomic expression and activity are governed by multiple regulatory mechanisms. Protein stability and degradation, posttranslational modifications, and protein complex formation are among the processes that often make gene expression profiles disappointing surrogates for explaining cellular function. Previous studies in colon, breast, and ovarian cancers have shown that protein abundance cannot be reliably predicted from gene expression measurements.^13–15^ Direct measurement of protein markers, however, has proven to be robust and reliable prognostic and theranostic tools in many cancer types (eg, HER2-neu, ER, and PR in breast cancer), which has generated significant interest in proteomics within the glioma field. Proteomic analyses have identified differences in protein profiles between high- and low-grade gliomas,^16,17^ between different molecular subtypes of gliomas,^18^ between glioma patients who are chemosensitive and those who are not,^19^ between GB and normal brain tissue,^20,21^ between different-grade tumor areas in the same patient,^22^ and between proteins that are exclusively expressed by GB and those that are not.^23,24^ We have previously reported that protein network classifiers can predict GB patient survival independent of age or gene expression subtype^25^ and used reverse-phase protein array data to construct a prognostic GB protein signature.^26^ However, these studies have been limited in that they have either interrogated a small number of proteins, used very small GB patient sample sizes, or utilized cell lines. The Cancer Genome Atlas (TCGA), for example, on which we based our previous work, examined only 171 proteins.^27^

Technological advances in liquid chromatography (LC) and mass spectrometry (MS) suitable for high-throughput protein profiling coupled with standardization of GB tissue banking set the stage for our current study. In this study, we paired LC and tandem MS (LC-MS/MS) to identify and verify novel prognostic candidate protein markers in GB not anticipated from previous genomics studies. We verified our findings in an independent proteomics dataset. We also assessed the correlation between protein expression and mRNA expression from data generated from the same discovery GB tumors (ie, analyzing paired LC-MS/MS data and RNAseq data). Additionally, we have used label-free proteomics to examine how protein abundance varies throughout the tumor using an independent multisample dataset. We anticipate that making publicly available our matched proteomic and RNAseq data as well as the multisample proteomics dataset will allow other researchers to make novel observations regarding their proteins of interest.

## Methods

### The Ohio Brain Tumor Study Population

**Figure 1.**
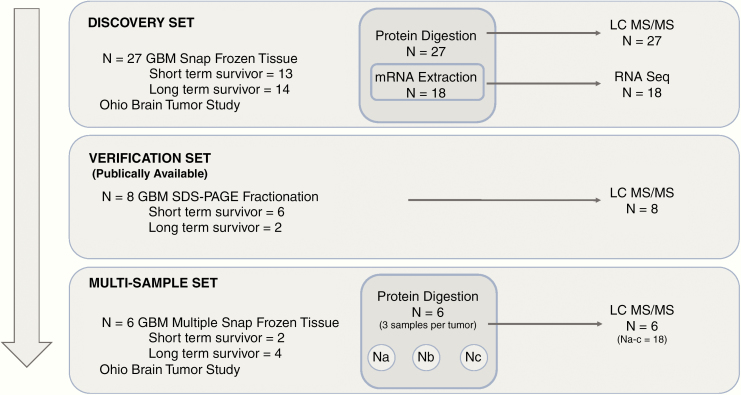
Experimental schematic for discovery, verification, and multisample datasets. In the discovery dataset protein and mRNA were isolated from 27 snap-frozen GB samples from the Ohio Brain Tumor Study, RNAseq and LC-MS/MS were performed on the samples. In the publicly available verification dataset protein was extracted using SDS-PAGE fractionation and quantified using LC-MS/MS from 8 snap-frozen GB samples. Finally, using a multisampling approach 3 distinct samples were taken from 6 different GB patients and a total of 18 samples were quantified using LC-MS/MS.

Newly diagnosed untreated GB patients were prospectively recruited at University Hospitals Cleveland Medical Center under the Ohio Brain Tumor Study (OBTS) Institutional Review Board approved protocol^28^; all patients provided written consent for participation in OBTS. We obtained snap-frozen tumor samples, from each patient, in the operating room within 15–30 min post-resection using our established OBTS standardized operating procedures (SOPs). Our SOPs align with TCGA procedures for frozen tumor tissue ensuring reliable analyte extraction and molecular characterization.^27^ From each patient, we also conducted a medical chart review, including complete treatment information and active yearly follow-up for clinical outcomes. We defined short-term survivors (STS) as less than or equal to 10 months post-diagnosis and long-term survivors (LTS) as at least 18 months post-diagnosis, representing the 25% and 75% percentiles, respectively, of the overall OBTS study population survival distribution (*N* > 300). All samples were reviewed and annotated by an expert neuropathologist (M.E.C.) with regard to location and tumor cell and extent of necrosis concentration (M.E.C.). In addition, all patients included in this study received standard therapy, surgical resection followed by concurrent radiation and temozolomide. In our *discovery* set we used snap-frozen tumor samples from 13 STS and 14 LTS patients (total *N* = 27). The *multisample* dataset included 18 snap-frozen samples from 6 patients (3 samples by 6 patients) not included in the discovery set. In this dataset 3 distinct samples were taken from each patient and annotated as to tumor location by the neurosurgeon in the operating room: solid tumor, infiltrated brain, enhancing margin, or necrotic core. All tumor samples were centrally reviewed by a board-certified neuropathologist (Figure 1).

### Protein Recovery From Snap-Frozen GB Patient Samples From OBTS

All snap-frozen tumor samples were processed in the Center for Proteomics and Bioinformatics using previously published procedures, summarized here.^25^

Liquid chromatography–mass spectrometry analysis (LC-MS/MS) and data processing from GB patient samples from OBTS.

The digests prepared from snap-frozen tumor samples (600 ng protein load for discovery dataset) were randomized and analyzed by an LC-MS/MS system using a Waters NanoAcquity Ultra Performance Liquid Chromatography system (Waters) that was interfaced to a Linear Ion Trap Quantization Elite-Orbitrap mass spectrometer (Thermo Finnigan). The platform was operated in the nano-LC mode using the standard nano-electrospray ionization, atmospheric pressure ionization stack fitted with a 360 uM × 20 uM picotip emitter (New Objective). The solvent flow rate through the column was maintained at 300 nL/min. The protein digests were injected onto a reversed-phase symmetry C18 trapping column (0.18 × 20 mm, 5 µm particle size, Waters, Inc.) equilibrated with 0.1% formic acid (FA)/2% acetonitrile and washed, bound peptides were chromatographed using a linear gradient of acetonitrile from 5% to 50% in aqueous 0.1% FA over a period of 210 min. A 100% acetonitrile elution step was performed for 15 min prior to resetting the analytical column to the initial equilibration conditions and for 15 more minutes at the end of the chromatographic run, accounting for a total of 240 min of LC-MS/MS run time. The mass spectrometer was operated in a data-dependent MS to MS/MS switching mode, with the 20 most intense ions in each MS scan subjected to MS/MS analysis. The full scan was performed at 60 000 resolution in the Orbitrap detector and the MS/MS fragmentation scans were performed in the dual ion trap detector collision-induced dissociation mode such that the total scan cycle frequency was approximately 1.5 s. The dynamic exclusion function for previously selected precursor ions was enabled during the analysis such that the following parameters were applied: repeat count of 2, repeat duration of 45 s, exclusion duration of 60 s, and exclusion size list of 450. Xcalibur software (version 2.0.7, Thermo-Finnigan Inc.) was used for instrument control, data acquisition, and data processing. In order to monitor LC/MS/MS reproducibility across individual sample analyses, 400 fmol of external heavy labeled peptides from a calibration mixture (part number 88321 Thermo Scientific) was used. Overall an average ±2.0 min drift in retention time was observed for QC peptides with an average of coefficient of variation in peptide intensities of 23% across the samples. Raw LC/MS and LC/MS/MS MS spectra from the GB tumor samples were processed using Rosetta Elucidator as previously described.^29,30^ An unfractionated differential label-free analysis, with STS and LTS as the classifier groups for quantification using the chromatographic peak volume, was used. Feature definition and peak identification of the aligned data were done according to nominally accepted PeakTeller parameters.^30^ Data (*.DTA) files were created from this workflow, and the data were exported to a MASCOT search engine (http://www.matrixscience.com/) for database searching on the International Protein Index using the UniProt website (http://www.uniprot.org/). Annotated features were further processed via ProteinTeller, using previously documented parameters.^30^

### Independent Verification Dataset From GB Patient Samples From the Mirza Laboratory

All snap-frozen GB samples in the HerouxMirza dataset were processed at the Medical College of Wisconsin, in the Mirza laboratory, using sodium dodecyl sulfate–polyacrylamide gel electrophoresis (SDS-PAGE) fractionation followed by LC-MS/MS. The proteomics methods and bioinformatics pipeline have been previously published.^21^ Only samples from patients with OS less than or equal to 10 months of at least 18 months were included in this study (*N* = 8 total).

### Data Processing and Analysis

For all data generated on GB patients from OBTS, peptide peak intensities were normalized using an adaptation of surrogate variable analysis designed for MS data, whereby singular value decomposition is executed on model residuals in order to identify bias trends.^31,32^ Normalization of peptide intensities was implemented using the ProteoMM R package.^33^ Missing values accounted for less than 1% of all peptide peak intensities in the discovery dataset and were not imputed.^34^ Relative protein abundance was calculated by averaging the top 3 most abundant peptides per protein.^35,36^ Further downstream analysis was then conducted using relative protein abundance. *Z*-scores were generated for all proteins and used for unsupervised and supervised hierarchical clustering analyses.

Differential expression analysis between STS and LTS was performed on both the OBTS dataset and HerouxMirza datasets using the empirical Bayes method executed with the *limma* R package.^34,37^ RNA sequencing data were processed, normalized, and analyzed as previously published.^38^ Following log2 transformation, the correlation between protein abundance and gene expression counts was assessed using Pearson’s correlation.

In the multisample dataset, the variance of a protein was defined as:

$$\begin{array}{l} s^{2}=\frac{\sum {\left(x\;\;\;-\;\;\;\overset{-}{x}\;\right)}^{2}}{N-1}= \end{array}$$

where *x* is the abundance of the protein in a patient’s sample, is the mean abundance of the protein across all of the patient’s samples, and *N* is the number of total samples for the patient. Pearson’s correlation coefficient was also used to measure the strength of the linear association between LC-MS/MS samples from the same patient in the multisample dataset.

All analyses were completed in R version 3.3.3 (http://www.R-project.org).

Gene set enrichment of gene ontology functional groups was conducted using Gene Set Enrichment Analysis (GSEA).^39^

### Dataset Availability

Unnormalized peptide level data for the discovery and multisample datasets are included as Supplementary Tables S1 and S2. The RNA sequencing data from the same patient group as the discovery proteomics dataset are available from the EMLBL-EBI European Nucleotide Archive database with accession number PRJEB10881 and is accessible via http://www.ebi.ac.uk/ena/data/view/PRJEB10881. The sample accession numbers from ERS848749 to ERS848765 are for RNA sequencing. The RNA sequencing methods, processing details, and differentially expressed gene lists are available here.^38^ The HerouxMirza proteomics dataset and methods have been previously published.^21^ Seventeen of the OBTS patients for whom we have generated proteomics data were also included in TCGA. The TCGA IDs for these patients are listed in Supplementary File 3 and TCGA omic data can be downloaded from the Genomic Data Commons Data Portal (http://www.portal.gdc.cancer.gov).

## Results

Our discovery dataset was compromised of 27 patients, including 13 short-term survivors (STS; OS ≤10 months) and 14 long-term survivors (LTS; OS ≥18 months). For patients included in our discovery study, age at diagnosis, *IDH1/2* mutational status, and *MGMT* methylation status were similar among STS and LTS (for those who were tested for *IDH1/2* mutation and *MGMT* methylation; some patients were diagnosed prior to the current standard of testing for these biomarkers) (Table 1). The LTS group had more individuals with KPS score at least 70. All patients, except for one, received standard therapy (concurrent radiation and temozolomide) (Table 1). Similar characteristics were seen in the multisample and HerouxMirza datasets (Table 1). All patient samples in the discovery and multisample datasets passed quality control. Extended clinical data for the discovery and multisample datasets can be found in Supplementary Table S3.

**Table 1.** Hallmark Clinical Characteristics of 3 Independent GB Study Datasets

	Discovery Dataset		HerouxMirza Dataset		Multisample Dataset	
	STS (*N* = 13)	LTS (*N* = 14)	STS (*N* = 6)	LTS (*N* = 2)	STS (*N* = 4)	LTS (*N* = 2)
Median age at diagnosis (range)	58 (39–77)	61 (48–83)	64.5 (53–67)	56.5 (48–65)	67.5 (64–74)	59 (54–64)
Median overall survival (months) (range)	5.98 (3.37–10.12)	25.59 (19.70–66.51)	4.77 (1.27–9.70)	28.09 (20.47–35.70)	6.51 (1.65–7.34)	30.87 (18.50–43.24)
Male (*N*)	11	5	2	1	2	1
Concurrent radiation and temozolomide (*N*)	12	14	NA	NA	1	2
IDH mutation (*N*)	1 (NT = 6)	1 (NT = 8)	NT	NT	0 (NT = 2)	0 (NT = 2)
MGMT methylation (*N*)	5 (NT = 5)	5 (NT = 6)	0	1	1 (NT = 2)	0 (NT = 2)
KPS ≥ 70 (*N*)	3 (missing = 3)	11 (missing = 3)	4 (missing = 2)	0 (missing = 2)	0 (missing = 2)	1 (missing = 1)

NT, not tested; NA, not available.

### Pathway Protein Dysregulation in STS Versus LTS GBs

**Figure 2.**
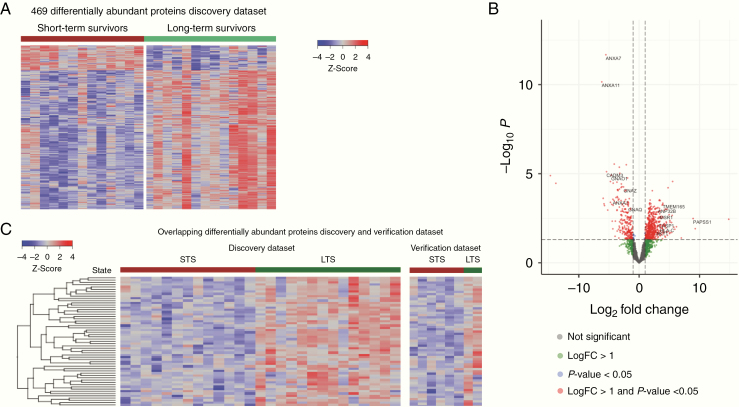
(A) Heatmap shows the *z*-score for the 469 proteins that were significantly differentially abundant (FDR *P*-value < .05) between STS and LTS in the discovery dataset. Individual patient samples are in columns. A patient’s survival (STS, OS ≤10 months or LTS, OS ≥18 months) is indicated by color-coded labeling in horizontal bars above the heatmap. (B) Volcano plot shows differential protein abundance between STS and LTS in the discovery dataset, where −logFC indicates increased protein abundance in STS and +logFC indicates increased protein abundance in LTS. Each dot represents a protein and they are color coded as follows: gray (not significant), green (logFC >1), blue (*P* < .05), and red (logFC > 1 and *P* < .05). (C) Heatmap shows the *z*-score for the 67 proteins that were significantly differentially abundant (FDR *P*-value < .05) between STS and LTS in the discovery dataset and verification dataset. Individual patient samples are in columns. A patient’s survival (STS, OS ≤10 months or LTS, OS ≥18 months) is indicated by color-coded labeling in horizontal bars above the heatmap.

Using LC-MS/MS, we identified 11 877 peptides in 2495 unique proteins in our discovery samples. Unsupervised hierarchical clustering showed that LTS and STS groups could be readily distinguished by protein abundance (Supplementary Figure S1). We identified 469 proteins that were differentially abundant between STS and LTS (Welch’s *t*-test, FDR *q* < 0.05; Figure 2A, Supplementary Table S4). Of the significantly differentially abundant proteins, 393 were upregulated in LTS and 76 were upregulated in STS (Figure 2A). Gene ontology functional group analysis demonstrated that STS were enriched in proteins involved in neuronal and axon development, cytoskeleton organization, and cell adhesion and signaling (Fisher’s exact test, FDR *q* < 0.05; Supplementary Table S5). LTS were enriched in proteins involved in RNA binding and catabolism, and protein localization, targeting, and transport (Fisher’s exact test, FDR *q* < 0.05; Supplementary Table S5).

Proteins that were significantly (*P* < .05) upregulated in STS with a logFC less than −1 included calcium-dependent phospholipid-binding proteins from the annexin family (ANXA1/2/4/7/11; Figure 2B). ANXA1/2 has been shown to be regulators of p53 signaling.^40–43^ Additionally, proteins (GNAO1, GNAZ, GNAQ, and DNM1) involved in PAR-1-mediated thrombin signaling were upregulated in STS (Figure 2B). PAR-1 and thrombin signaling are currently being investigated as therapeutic target in GBs.^44^ In LTS we observed a diverse array of proteins with significantly (*P* < .05) increased abundance (log-fold change >1) that included HLA-C an important activator of immune response; CASP1 a member of the p53 signaling pathway apoptotic pathway; and surprisingly AKT1 a critical oncogenic regulator of apoptosis (Figure 2B).

Using a previously published independent GB dataset we sought to verify the proteins we identified as differentially abundant between STS and LTS. The verification dataset contains 6 STS and 2 LTS GB samples that were interrogated using SDS-PAGE fractionation followed by LC-MS/MS. We compared the significantly differentially abundant proteins in the discovery dataset to the verification proteomics dataset. We found that of the 469 proteins significantly differentially abundant in the discovery dataset, 67 were also significantly differentially abundant in the verification GBs (Figure 2C; Supplementary Table S6). The 67 proteins, which were differentially abundant both datasets, were significantly enriched in proteins involved in axon guidance, such as ribosomal proteins (RPS3A/11/23 and RPL4/7/8/15), 26S proteasomes (PSMD11/13), RHOB, ACTR2, CNTN1, and DPYSL2. Additionally, there was enrichment in WNT signaling proteins (AKT1, HIST1H2AE, HDAC1, RUVBL1, and PSMD11/13), interferon-gamma response (NUP93, ADAR, STAT3, PTPN6, TRIM25, and CD74), and cytokine response (DHX9, ADAR, HNRNPF, TRIM25, AKT1, STAT3, CD74, MYO1C, ACTR2, PSMD11/13, PTPN6/12, PYCARD, and CD47).

### Gene Expression Does Not Reliably Predict Protein Abundance in GBs

**Figure 3.**
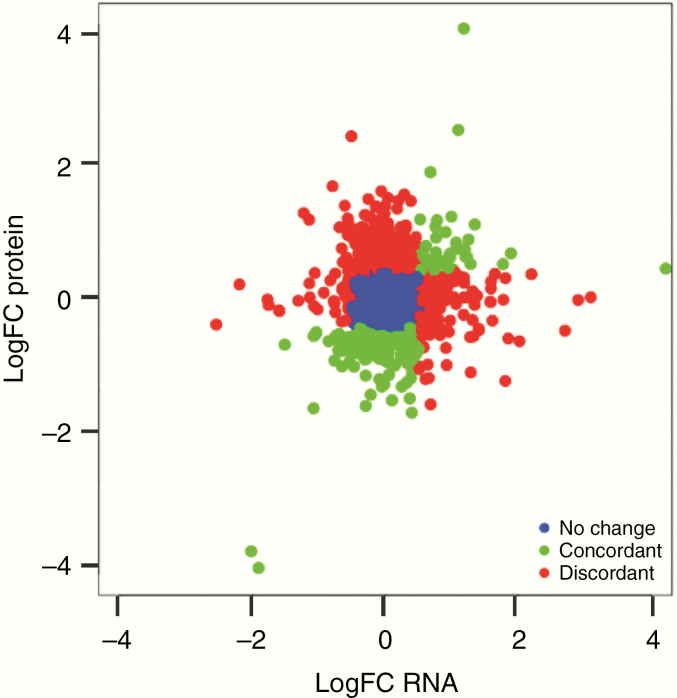
Scatter plot of logFC values differential expression in the discovery dataset for both RNAseq and protein abundance. For 2369 gene/protein pairs the logFC in the RNAseq data is plotted versus the logFC in the protein LC/MS-MS data. Each dot represents a gene/protein and they are color coded relative to the correlation between protein abundance changes and gene expression changes, where blue is no change (−1 < logFC < 1 for both datasets), green is concordant (logFC < −1 or logFC > 1 for both datasets), and red is discordant (logFC < −1 for protein and logFC > 1 for gene expression or vice versa).

Matched proteomic and RNAseq data from the discovery GB samples allowed the first whole-exome analysis of transcript–protein relationships in GB. We compared the abundance of identified proteins with the corresponding mRNA abundance for each patient sample. All samples showed significant positive mRNA–protein correlation (FDR-adjusted *P*-value < .0001, Pearson’s correlation coefficient) with an average correlation between protein and mRNA abundance of 0.22 (Table 2). This result is consistent with previous studies in ovarian, colorectal, and breast cancers (correlation coefficients ranging from 0.23 to 0.45).^13–15^ In addition to the overall patient-level correlation between protein abundance and mRNA, we examined the correlation between mRNA and protein abundance at the gene/protein level. There were 2369 genes/proteins for which there were both mRNA and protein measurements available. We found that only 55% of these genes showed a positive mRNA–protein correlation (Supplementary Table S7; Figure 3). We compared proteins that were significantly differentially abundant (LC-MS/MS data) between STS and LTS to the genes that were significantly differentially expressed (RNAseq data) in the same patients. Of the 469 significantly differentially abundant proteins and the 615 significantly differentially expressed genes between STS and LTS, we found only 7 corresponding gene/protein pairs overlapping between the 2 lists (SUB1, PSMB8, CADM3, GNG7, RPL23, PDIA4, and PSMB9).

**Table 2.** Patient-Level mRNA–Protein Pearson’s Correlation for the Discovery Dataset

Patient ID	*P*-value	Pearson Correlation Coefficient
LTS_1	1.43E-23	0.20
LTS_2	3.74E-19	0.18
LTS_4	1.47E-21	0.19
LTS_5	1.92E-23	0.20
LTS_6	6.79E-27	0.22
LTS_7	2.17E-24	0.21
LTS_8	2.60E-22	0.20
LTS_9	1.61E-31	0.24
LTS_10	3.66E-18	0.18
LTS_11	1.37E-27	0.22
LTS_12	1.90E-32	0.24
LTS_13	2.87E-35	0.25
LTS_14	1.11E-18	0.18
STS_1	2.43E-26	0.22
STS_2	4.33E-30	0.23
STS_3	3.02E-30	0.23
STS_5	1.08E-45	0.29
STS_6	4.95E-28	0.22
STS_7	1.81E-20	0.19
STS_11	7.84E-35	0.25

We next examined whether the correlation between protein and mRNA variation was related to the biological function of the gene/protein by performing a GSEA using the set of 2369 gene/protein pairs that had suitable mRNA and protein measurements available. Consistent with previous analysis in other cancer types, genes involved in metabolic processes (amino acid, lipid, and sugar metabolism) had high concordance between mRNA and protein abundance (correlation coefficient >0.25).^13–15^ Additionally, specific to GB we also found high concordance among gene/protein pairs involved in cytokine and immune system signaling. Also consistent with previous work, we found a negative correlation between gene/protein pairs involved in pathways such as mRNA splicing, spliceosome machinery, and protein translation and posttranslational modifications. Additionally, in the GB dataset we found a low correlation among cell cycle, axon guidance, and cellular response to stress gene/protein pairs.

### Protein Heterogeneity Exists in GBs by Survival Group, Location, and Intratumorally

**Figure 4.**
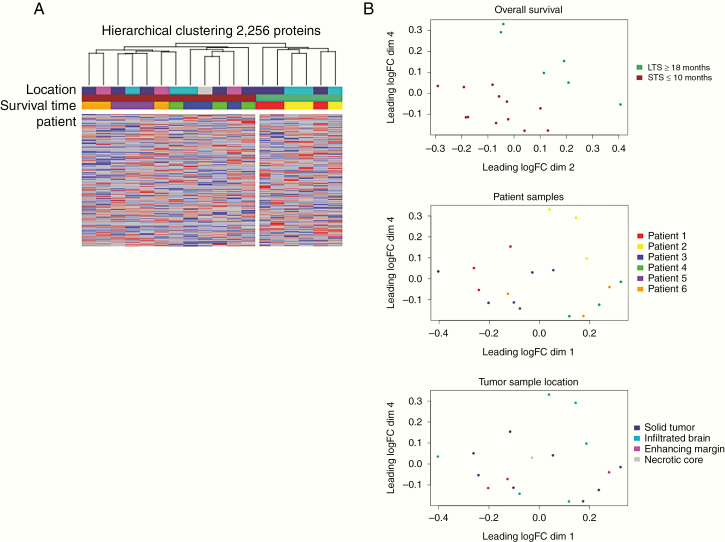
(A) Hierarchical clustering of GB samples in the multisample dataset using *z*-score of 2256. Samples are in columns and are color coded based on the patient, patient’s overall survival (STS, OS <10 months or LTS, OS >16 months), and tumor sample location (solid tumor, infiltrated brain, necrotic core, or enhancing margin). (B) Multidimensional scaling plot shows the distribution of samples from the multisample dataset based on the patient’s overall survival (top), patient sample (middle), and tumor sample location (bottom).

In addition to the disconnect between mRNA expression and protein abundance, intratumoral heterogeneity has hampered the development of reliable biomarkers and targeted treatments in GB. To that end, we sought to additionally create a dataset that initially assessed how protein abundance varied throughout the tumor. Using LC-MS/MS, we identified 2256 proteins from an independent set of 18 GB samples (6 patients total, 3 samples from each patient) (Supplementary Table S2). Unsupervised hierarchical clustering demonstrated that samples from STS and LTS, respectively, clustered together (Figure 4A). Samples from the same patient demonstrated a high degree of similarity with one another overall (Figure 4B; Pearson’s *r*; Patient 1: 0.85–0.88, Patient 2: 0.87–0.89, Patient 3: 0.85–0.87, Patient 4: 0.85–0.87, Patient 5: 0.83–0.89, Patient 6: 0.85–0.87). However, the variance in protein abundance by protein among intratumoral samples was high (Supplementary Table S8; Patient 1: median 0.14, range 0–6.17; Patient 2: median 0.12, range 0–8.67; Patient 3: median 0.15, range 0–8.98; Patient 4: median 0.17, range 0–6.27; Patient 5: median 0.17, range 0–5.88; Patient 6: median 0.18%, range 0–7.88). Tumor sample location (solid tumor, infiltrated brain, enhancing margin, or necrotic core) had minimal impact on cluster membership (Figure 4B). More than 40% of the proteins identified in our 6 patients had intratumoral variances in abundance greater than 0.25. Housekeeping proteins such as beta-actin, GAPDH, and VCP were among the most homogenously expressed within and across our patient samples (Supplementary Table S8). While important cancer drivers such as TGFB1 and KRAS exhibited wide intratumoral expression (Supplementary Table S8).

## Discussion

In this study using snap-frozen tumor samples from GB patients receiving standard therapy, we identified potential GB proteins that were significantly differentially abundant between STS and LTS. Notably, we have generated 2 distinct unlabeled GB proteomics datasets. First, we generated a matched proteomic and RNAseq dataset that allowed for the first whole-genome analysis of transcript–protein relationships in GBs. Then, we verified the proteins most differentially abundant between STS and LTS in an independent dataset. Finally, we generated an unprecedented dataset of proteomic data from multiple regions in each (of 6) patient’s tumor allowing for preliminary investigation of intratumoral heterogeneity. One of the daunting challenges facing modern medicine lies in the understanding and treatment of heterogeneous tumors. The complexity of protein profiles from tumors must be characterized, compared, and annotated with clinical outcomes in order to develop more effective therapeutic strategies; however, high-throughput proteomics has until now been underused in GB research. Here we make publicly available a multisample dataset that will allow researchers to begin to assess the heterogeneous distribution of their proteins of interest.

In this study, we have applied high-throughput shotgun proteomics to study independent sets of GBs in order to identify proteins that are differentially abundant among STS and LTS. Comparison of our proteomics data with gene expression data from the same patient set demonstrated a general departure from the expected relationship between RNA expression and protein abundance. Few of the major proteins that correlated with survival in our proteomics analysis showed corresponding correlation using RNAseq data. Only 6 protein/gene pairs were significantly differentially expressed in both the proteomics and RNAseq data from the same patients. This result suggests that protein regulatory mechanisms are disrupting the correlation between gene expression and protein abundance in GBs. This finding further illustrates the important role of proteomics in identifying the dysregulation of cell processes that may be missed by expression-based approaches.

Using proteomics, we identified and independently verified a set of potential biomarkers and drug targets as differentially abundant between STS and LTS. For example, proteins involved in axon guidance (ribosomal proteins [RPS3A/11/23 and RPL4/7/8/15], 26S proteasomes [PSMD11/13], RHOB, ACTR2, CNTN1, and DPYSL2) were significantly enriched in STS in both our discovery and verification datasets. Cell movement along white matter tracts, guided by axonal guidance proteins, is a well-known route of glioma cell invasion.^45^ This finding also validates our previous work using RNAseq and a smaller independent GB proteomics dataset *n* = 16, which found that PSMD3 was part of a network protein signature that predicted GB patient survival with more than 80% accuracy.^46^ In contrast, BAX, CASP1, GNB2L1, and VWA5A provided core p53 signaling enrichment in LTS. Additionally, we observed the dysregulation of calcium-dependent phospholipid-binding proteins from the annexin family (ANXA1/2/4/7/11). These proteins play important roles in a variety of processes, including cell signaling, proliferation, differentiation, and apoptosis. Importantly, both proteins are frequently deregulated in many cancers, though there are highly contrasting patterns of overexpression and downregulation reported depending on the tumor type.^47–53^ There are also reports that ANXA1/2 can function as tumor suppressors and that a decrease in expression can lead to drug resistance. In particular, ANXA2 has been implicated in regulating mesenchymal transformation in GB^54^ and has been correlated with GB tumor aggressiveness and grade,^55^ as well as GB grade and prognosis.^55,56^ Inhibition of ANXA2 in GBs has been shown to dramatically impair cell migration.^55^ In addition, a previous study showed that the glioma protein profile varied significantly by *IDH1/2* mutation and 1p/19q deletion status18; however, in the present study we did not have GB samples with differences in *IDH1/2* mutation status by patient survival groups in order to address this question (and 1p/19q deletion is a hallmark feature for non-GB tumors).

Here we have shown how unbiased proteomic technologies can be harnessed to better understand GB vulnerabilities. Due to the cost-prohibitive nature of the work presented here, our work is limited by relatively small sample size, however, it is significantly larger than any cohort previously published. Our work will benefit from future expansion in terms of both sample size and workflow in order to establish the robustness of identified proteins as prognostic markers or potential drug targets. In addition, we anticipate that the proteins and subsequent biomarkers we have detected to be of higher abundance. While the proteins that we have quantified are more highly abundant overall we still find that even these highly abundant proteins do not always closely correlate with mRNA expression. In fact, in this study we have demonstrated that there is a significant disparity among protein abundance and mRNA expression. While a strength of our study was the inclusion of both RNAseq and proteomics measurements, future work would benefit from the inclusion of important phospho-proteomics data and the use of unbiased proteomics platforms that capture data from a larger number of proteins. We have also left unresolved any recommendation on the number of biopsies or sections required from tumors in order to fully assess GB protein heterogeneity. Finally, work correlating patient response to treatment directly to proteins and protein heterogeneity in a clinical trial setting would elucidate the scope of this issue as it relates to improving GB patient treatment and outcome. Ultimately, our approach combined with future work may be useful in pinpointing new drug targets, identifying drug response biomarkers, and stratifying patients into treatment groups. In conclusion, we make available to the glioma research community 2 independent, clinically annotated, shotgun proteomics datasets—one of which includes matched RNAseq measurements. This work forms the foundation for the identification of prognostic biomarkers, targeted molecular treatments, and patient stratification strategies for GB.

## Supplementary Material

vdaa039_suppl_Supplementary_Figure

vdaa039_suppl_Supplementary_Tables
