# Supplementary figures and images for: Proteins inform survival-based differences in patients with glioblastoma
Source: Neurooncol Adv. 2020 Mar 17;2(1):vdaa039. doi: 10.1093/noajnl/vdaa039 (PMC7212893; doi:10.1093/noajnl/vdaa039)

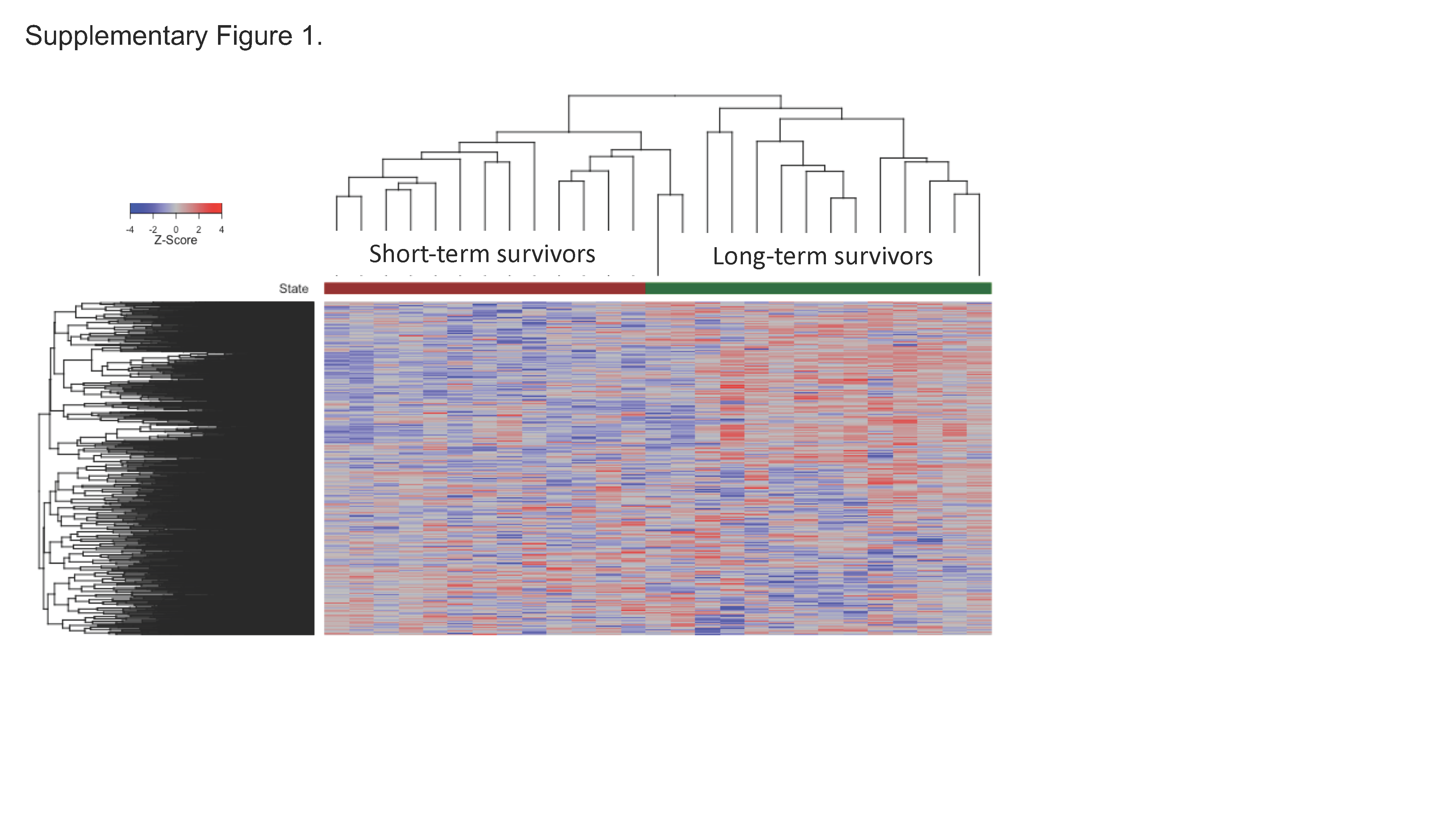

Supplement: vdaa039_suppl_Supplementary_Figure [file vdaa039_suppl_supplementary_figure.png]
